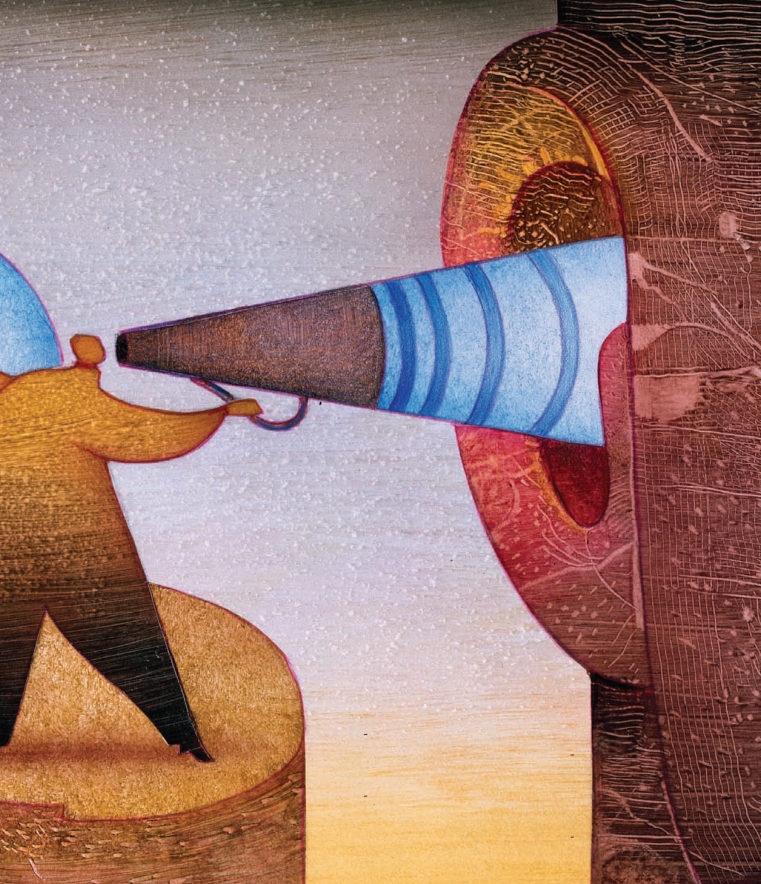# Communication Gap: The Disconnect Between What Scientists Say and What the Public Hears

**DOI:** 10.1289/ehp.117-a548

**Published:** 2009-12

**Authors:** Charles W. Schmidt

**Affiliations:** **Charles W. Schmidt**, MS, of Portland, Maine, has written for *Discover Magazine*, *Science*, and *Nature Medicine*. In 2002 he won the National Association of Science Writers’ Science-in-Society Journalism Award

Mojib Latif probably didn’t anticipate the public reaction his research would attract last year. Writing in the 1 May 2008 issue of *Nature*, he and his colleagues from the Leibniz Institute of Marine Sciences and the Max Planck Institute in Kiel, Germany, predicted that increases in mean global temperatures could pause into the next decade, even though greenhouse gas levels were still rising in the atmosphere. That lull in warming, their models showed, was temporary, and due to complex interactions between the atmosphere and periodic cooling cycles in the oceans.

A meteorologist and oceanographer, Latif emphasized that these cyclical variations could occur even in the face of long-term climate trends. But to his surprise, skeptics seized on the findings as evidence that mean global temperatures aren’t really rising. The website newsbusters.org, for instance, which bills itself as “dedicated to documenting, exposing, and neutralizing liberal media bias,” compared Latif’s findings to “the Pope suddenly [announcing] the Catholic Church had been wrong for centuries about prohibiting priests from marrying.” To Latif, the implication that climate change is a hoax was preposterous. “Making inferences about global warming from my short-term climate prediction is like comparing apples and oranges,” he says.

Latif was caught in a familiar media trap. Research often delivers statistically nuanced findings that the lay public as well as journalists and other science communicators can find hard to understand. And just as political messages can be twisted into snippets of misinformation, scientific findings, too, are vulnerable to distortions and misrepresentations that stick in the public mind, especially if they fit ideologic biases.

These distortions are becoming all too common in today’s new media environment. Although the World Wide Web offers invaluable access to information, it also gives an audience to anyone with an ax to grind. According to a commentary in the June 2009 issue of *Nature Biotechnology* authored by 24 experts in communication, law, and journalism, media fragmentation and the rise of ideologically slanted websites are perpetuating gridlocked opinions in science, just as they are in politics.

One of those authors is Matthew Nisbet, an assistant professor of communication at American University in Washington, DC. He says people who aren’t inclined to pay close attention to an issue will learn about it from media outlets that reinforce their own social, political, or religious views. This and other types of “mental shortcuts,” he says, make it possible for individuals to draw quick conclusions about complex topics that fit their own preconceptions.

Given these trends, communication experts are calling for fundamental changes in how scientists interact with the media because debates over climate change, health, energy, and technology are simply too important to lose to misinformation. As always, scientists are encouraged to communicate clearly using language that nonspecialists can understand. But now they’re also being urged to step beyond the confines of the laboratory and to become more engaged in efforts to educate the public.

“The ultimate goal [in science communication],” says Nisbet, “is civic education—enabling and motivating more people into thinking, talking, and participating in collective decisions about, for example, what to do about climate change, or how to fund and oversee biotechnology.” Scientists need to somehow communicate scientific uncertainties while going head-to-head against oversimplified inaccuracies in the media. The question is how best to do that.

## Reworking the Angle

Nisbet in particular seeks to move beyond the traditional “deficit model” that currently dominates science communication. The deficit model assumes that if nonspecialists only understood the scientific facts, they would see eye-to-eye with the experts. Ignorance is what drives controversies in science, the model postulates. And by filling that deficit with knowledge, scientists can help make these controversies disappear.

But does that assumption really hold true? Not necessarily, Nisbet says. Disputes over climate change, for instance, remain strong despite the sustained efforts of scientists to communicate about the issue through the media. An October 2009 survey by the Pew Research Center for the People & the Press suggests public opinions about climate change line up more on political than scientific grounds.

According to that survey, 75% of Democrats see solid evidence that the average temperature on Earth has been getting warmer over the past few decades, compared with just 35% of Republicans. That disparity, Nisbet says, reflects opposing media influences geared toward their respective audiences. Both Republicans and Democrats tend to rely on news outlets that affirm their own social values, he says. And those outlets—together with input from like-minded friends and colleagues—can be more influential than the science itself.

Tellingly, the Pew survey also indicates that, compared with survey responses from April 2008, 8% fewer Democrats and 14% fewer Republicans reported seeing solid evidence of warming, which suggests confidence in the research is declining across party lines. The surveyors do not comment, however, on the reasons for that decline or whether it might reflect contradictory coverage of climate change in the press.

Nisbet is well known for his research on framing, or defining scientific issues in ways that audiences can understand in part by appealing to their core values. Climate change skeptics already do this successfully by predicting economic doom from curbing greenhouse gas emissions, he says. “You need to use metaphors and narratives that make the issue personally relevant,” Nisbet explains. “It’s got to be understandable and interesting to audiences that don’t understand the technical details.”

Teaming with evangelical leaders has enabled some scientists to frame climate change in terms of religious morality, which helps to engage conservative Christians on the issue. Among them are Eric Chivian, director of the Center for Health and the Global Environment at the Harvard Medical School, and Richard Cizik, founder and president of the recently formed New Evangelicals, who famously joined forces in 2007 to educate law makers and the public about environmental threats. Cizik is quoted in the 18 November 2009 online edition of the U.K. *Guardian* as saying that younger generations of evangelicals in particular “have an intensity level that even some in the environmental community don’t have. They believe [environmental stewardship] is their God-given calling.”

But Sharon Dunwoody, a professor of journalism and mass communication at the University of Wisconsin–Madison, cautions that frames might be labeled as spin by audiences who feel they’re being manipulated. A climate change activist, for instance, might think it’s effective to frame climate change in terms of dying polar bears. But a skeptic who doesn’t think polar bears are at risk from climate change might feel manipulated by that frame and view it as spin.

To that, Nisbet says, “‘Spin’ is a problematic term since people use it in multiple ways and really never define what they mean by it. They usually just throw it out there as a way to express criticism without actually explaining what their criticism might be, or what their preferred alternative is.”

## Maintaining Credibility

Framing can pose other tough challenges for scientists; it requires them to know and understand what elements will engage a given target audience. And that begs insights into human nature that might not come readily to those more comfortable with data. Nisbet says talking points for use in framing can be obtained from research techniques familiar to social sciences research, such as interviews, focus groups, and surveys. Results from these investigations can be translated into practical advice for scientists who interact with different audiences via media formats such as web and video, he says.

Earl Holland, assistant vice president for research communications at The Ohio State University, argues that scientists are preoccupied with the day-to-day grinds of publishing and research, and therefore shouldn’t be obliged to consider public perceptions of their work so explicitly. He suggests, moreover, that those activities might compromise a scientist’s integrity.

Scientists often have the trust of the public going for them—they’re typically held in high esteem, Holland says. What elevates scientists over those who spread misinformation, he explains, is credibility, and that credibility lies in part on the notion that scientists make impartial judgments based on data. But when they align themselves with a particular side in a debate, that impartiality is put to the test, he says.

“As soon as scientists take up an advocacy role, regardless of the position or topic, they lose credibility as unbiased sources,” Holland asserts. “Some say that’s too much to ask, but I say that just like journalists have to rein in their own political beliefs when reporting, scientists have to avoid catering to policy arguments. They’re still highly regarded, but if they just get in there and punch it out with their opponents, they risk losing integrity.”

Holland’s view is that university news offices and what he describes as “support networks for the scientific community” bear responsibility for couching how research findings enter into policy debates—not the scientists themselves. That’s not a universal view, however; many scientists see no problem with advocacy, as long as it’s guided by expertise and experience.

Bruce Lanphear, a professor at BC Children’s Hospital and Simon Fraser University in Vancouver, British Columbia, says debates over whether scientists should get involved in policy are mostly semantic. “There’s a certain school of thought that our job as epidemiologists is simply to report results in journals while others translate those findings for the public—I don’t subscribe to that,” he says. “I view my job as also helping to translate findings in ways that don’t mislead the public but that also help people understand why something is important.”

Lanphear is best known for research that links low-dose exposure to lead and other toxicants to developmental effects in children. As a medical doctor, he says his efforts to raise awareness about industrial toxicants in commerce are consistent with the Hippocratic Oath. “Activism is a direct extension of what I was trained to do as a doctor,” he says. “I feel an obligation to present data in ways that prevent dangerous exposures in the population.”

Lanphear appears unfazed by charges of alarmism, and he acknowledges there remain many unanswered toxicologic questions about lead, pesticides, and other chemicals. But their known risks also compel regulatory changes to minimize exposure, he says. In communicating about low-dose chemical risks, Lanphear aims to create a sense of urgency, which he says is a prerequisite to environmental legislation.

“That’s what it comes down to: community outrage,” Lanphear says. “We knew lead was toxic as far back as 1909. Why did it take so long to restrict how we use it? Because of inertia, lobbyists, and the tax revenues it was generating. It took outrage and lawsuits to move the legislation. A sense of urgency holds feet to the fire.”

## Aiming for Clarity

People might look to science for clear-cut statements that can help them make decisions about their health and lifestyle, says Louis Guillette, Jr., a professor of biology at the University of Florida at Gainesville. But fields such as climate research, genomics, and toxicology are all grappling with enormous data sets and models that generate probabilistic instead of definitive findings. Most genetic tests, for instance, can’t accurately predict if someone will get a disease; they can only suggest that someone has perhaps a 15% chance of getting the disease under certain environmental conditions. Likewise, climate models can simulate temperature changes, but they can’t predict exactly where or when impacts will occur.

Individuals looking for clarity with respect to environmental threats might want a scientist to say, for instance, that a chemical will cause a specific effect at a precise real-world dose, but laboratory experiments don’t allow for that, adds Guillette. Instead, experiments deliberately exclude confounding factors such as age, sex, or hormonal status to isolate a single variable’s effect on a particular outcome. In the real world, these variables work simultaneously, along with a host of other chemical exposures, to produce effects that vary by individual.

It’s important to provide the public with a baseline context for understanding what’s meant by “risk,” experts say. For instance, it’s meaningless to say that family history of a disease makes a person 10 times more likely to succumb to that disease. It is clearer to say that if 1 in 100,000 people in the general population has the disease, then family history increases the risk to 1 in 10,000. That still may be a noteworthy difference—but perhaps not cause for undue alarm.

It’s also important to specify what groups are being compared when talking about changes in risk so it’s clear whether those changes are being described in absolute or relative terms. For example, consider preeclampsia, which affects an estimated 4% of pregnancies. If an environmental exposure increases the absolute risk of preeclampsia by 30%, that would mean going from 4% to 34%. In contrast, a relative increase of 30% would mean going from 4% to 5.2%.

All these statistical details make it impossible for scientists to speak in absolutes, so they communicate instead in terms of statistical probabilities that ideally apply under most real-world scenarios. Scientists take these nuances for granted, but they make a world of difference to anyone who has to intepret what new findings mean on a practical level. That’s an essential issue, because research must somehow reconcile data with society’s desire for clarity on scientific issues.

Joann Rodgers, senior advisor for science, crisis, and executive communications at Johns Hopkins Medicine and past president of the National Association of Science Writers, says environmental health findings are particularly hard to convey because, in addition to their complexity, they evoke emotional responses; climate change, pollution, and many other environmental threats affect millions of people. “Environmental issues give rise to a lot of activism,” Rodgers says. “We tend to see that also in other fields, but there seems to be an extraordinary dose of mythologizing and ranting about science in the environmental health realm.”

Dunwoody emphasizes that, as sources in the media, scientists get to decide what they’re going to say. But she adds they should also be insightful about how those messages are received, given the need to dispel misinformation in the public arena. “The way you portray something dictates the take-home messages people walk away with,” she says. “You’ve got to be careful.”

## Figures and Tables

**Figure:**
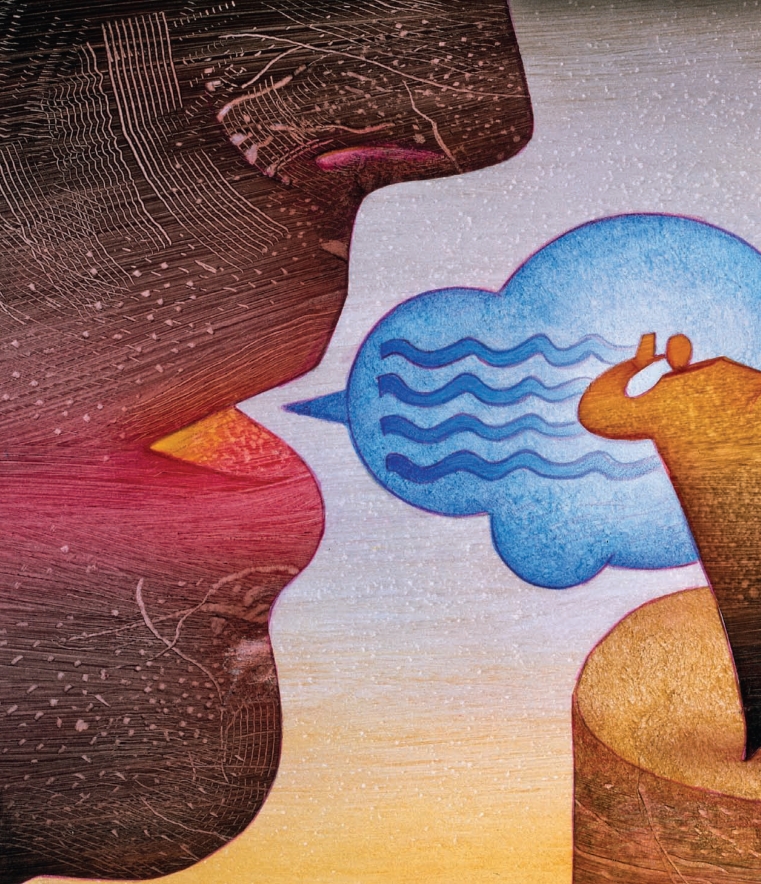


**Figure:**